# Complete genome sequence of *Staphylococcus epidermidis* B273 and its epidermicin NI01 biosynthesis plasmid

**DOI:** 10.1128/mra.00961-24

**Published:** 2025-01-10

**Authors:** Keir Nicholas-Haizelden, Charlotte E. Chong, Norah Alqahtani, Samah E. Alsaadi, Malcolm J. Horsburgh

**Affiliations:** 1 Institute of Infection, Veterinary and Ecological Sciences, University of Liverpool, Liverpool, United Kingdom; 2 Department of Genetics, University of Cambridge 2152 , Cambridge, United Kingdom; 3 Department of Biology, College of Science, Qassim University, Buradyah, Saudi Arabia; 4 Department of Biology, College of Science, Taif University, Taif, Saudi Arabia; Wellesley College Department of Biological Sciences, Wellesley, Massachusetts, USA

**Keywords:** *Staphylococcus*, epidermidis, bacteriocins

## Abstract

The human skin commensal *Staphylococcus epidermidis* produces diverse, therapeutically relevant bacteriocins. We report the complete whole-genome sequence of the nasal isolate *S. epidermidis* B273, which contains a plasmid with the biosynthetic gene cluster for epidermicin NI01, a broad-spectrum type II antimicrobial peptide.

## ANNOUNCEMENT

*Staphylococcus epidermidis* is a Gram-positive, coagulase-negative bacterium associated with lifelong colonization of human skin and nasopharynx (1, 2), which exerts a considerable healthcare burden via adhesion to indwelling medical devices (3). *S. epidermidis* isolates can produce bacteriocins and other antimicrobial peptides with therapeutic potential (4–8). *S. epidermidis* B273 contains pEMN1 with an epidermicin NI01 bacteriocin biosynthetic gene cluster (9). The complete genome sequence of *S. epidermidis* B273 will facilitate the study of therapeutically relevant epidermicin NI01, with its broad-spectrum activity (9).

*S. epidermidis* B273 was previously isolated using a healthy volunteer’s nasal swab serially diluted (0.9% saline [wt/vol]) onto nutrient agar (10). Strain B273 was cultured aerobically from a frozen glycerol stock on tryptone soy agar and then in broth (16–18 h, 37°C). DNA was purified using DNeasy Blood and Tissue Kit (Qiagen) according to the manufacturer’s protocol. Short-read Illumina sequencing was processed by SeqCenter (Pittsburgh, USA) using an Illumina DNA Prep Tagmentation Kit (20060059) and Illumina NovaSeqXPlus sequencer (https://www.seqcenter.com/). A coverage of 80× was obtained from 1,423,388 read pairs.

Oxford Nanopore long-read bacterial whole-genome sequencing was performed by Plasmidsaurus with DNA extraction using Zymo Quick-DNA Fungal/Bacteria Miniprep Plus Kit (https://www.plasmidsaurus.com/). Sequencing libraries were prepared using the DNA v14 ligation sequencing kit (SQK-LSK114) according to the manufacturer’s instructions with no size selection or shearing of DNA (Oxford Nanopore Technologies, Ltd, Oxford, UK). DNA libraries were sequenced using a PromethION P24 sequencer and FLO-PRO114M Flow Cell version R10.4.1 (Oxford Nanopore Technologies Ltd). Dorado for PromethION version 7.1.4 (Oxford Nanopore Technologies Ltd) was used to base call and demultiplex sequence reads. Total ONT reads (172,859) with a raw coverage of 300× and *N*_50_ of 6,849 (SeqKit) were obtained. Default parameters for bioinformatic tools were selected unless otherwise stated.

Illumina sequence reads were trimmed and filtered with Seqtk version 1.4 with default parameters (https://github.com/lh3/seqtk) and quality assessed with FastQC version 0.12.1 (https://github.com/s-andrews/FastQC). ONT sequence reads were trimmed via Filtlong version 0.2.1 (https://github.com/rrwick/Filtlong) to a minimum contiguous sequence cutoff length of 1,000 bp, a minimum mean quality threshold of 10, and removal of 5% lowest quality sequences. Trimmed ONT reads were downsampled to ~100× coverage via Rasusa version 2.0.0 (11). *De novo* long-read assemblies were generated via Flye version 2.9.4 (12). The resulting assembly was polished using long read sequences (Medaka version 1.12.1; https://github.com/nanoporetech/medaka) and short read sequences (Polypolish version 0.6.0) (13). Genomes were annotated using Prokaryotic Genome Annotation Pipeline version 6.8. Assembly quality and completeness were assessed via QUAST version 5.2.0 (14) and Busco version 1.0.0 (15), respectively. Plasmid contiguous sequences were identified using BLAST and PlasmidFinder version 2.1.1 (16, 17).

The whole genome sequence length of 2,523,268 bp has a GC content of 32% and final *de novo* coverage of 99.9×. PGAP annotation revealed 2,450 genes with 2,284 protein-coding sequences. The novel 38,555 kb plasmid, pEMN1, contains the epidermicin NI01 biosynthetic genes identified via BLAST and Clinker (17, 18). Three further plasmids were identified (23,220, 8,521, and 2,390 kb), showing high nucleotide homology to plasmids: pSP01 (accession_KR230047.1), plasmid 2 (accession_CP071998.1), and pAMT4 (accession_CP022252.1), respectively.

## Data Availability

The complete genome assembly of *S. epidermidis* B273 was deposited in the NCBI genome database under the RefSeq identifier GCA_041080605.1. Raw sequence reads from ONT and Illumina sequencing have been submitted to the Sequence Read Archive (SRA) and are available under the accession numbers SRR30107128 and SRR30107129, respectively.
